# On-farm approaches to increase whole-milk total solids: Effects on performance and health of dairy calves

**DOI:** 10.3168/jdsc.2024-0684

**Published:** 2025-03-12

**Authors:** Amanda M. Cezar, Ana Paula da Silva, Ariany F. de Toledo, Cristiane R. Tomaluski, Sophia C. Dondé, Gercino F. Virgínio Júnior, Marcos I. Marcondes, Carla M.M. Bittar

**Affiliations:** 1 Department of Animal Science, Luiz de Queiroz College of Agriculture, University of São Paulo, Piracicaba, 13418-900, Brazil; 2 Minas Gerais Agricultural Research Agency, Experimental Field of Montes Claros, Montes Claros, 39404-128, Brazil; 3 Department of Animal Science, Washington State University, Pullman, WA 99164

## Abstract

This study aimed to evaluate the performance, metabolism, and health of Holstein calves fed whole milk adjusted for 15% TS by adding a commercial solids corrector (245 g/kg CP, 48 g/kg crude fat, 90 g/kg of mineral, and 3 g/kg of ADF) using 2 practical approaches: daily adjustment based on a Brix refractometer reading, or the addition of a fixed amount of the corrector based on mean milk solids content. Thirty newborn male Holstein calves were assigned to one of the 2 treatments: (1) fixed, in which whole-milk solids were adjusted using a fixed dose of 25 g/L, and (2) Brix, in which whole-milk solids were adjusted based on a daily Brix refractometer reading. Treatments were randomly assigned within blocks formed based on BW and birth date (2 calves per block). The experimental period lasted 56 d, during which calves were fed 6 L/d of the respective liquid diet and had free access to water and starter concentrate. Daily adjustments based on the Brix refractometer reading improved total liquid diet, as well as protein and lactose intake. However, calf performance and health indicators did not differ between treatments. Total serum protein concentration was greater in calves fed milk corrected using the fixed dose. Additionally, creatinine concentration was influenced by the interaction between treatment and age, with a trend toward greater concentrations in calves fed milk adjusted using the daily Brix refractometer reading at 2 wk of age. Overall, both strategies for increasing TS in whole milk (Brix refractometer and fixed dose) resulted in comparable performance and health outcomes and may serve as practical on-farm approaches for managing dairy calves.

## Highlights

• On-farm approaches to increase the solids content in the liquid diet were evaluated.
• A fixed dose and adjustment by Brix refractometer had similar results in calf performance.
• Using a fixed dose of the product could facilitate management on dairy farms.

## Graphical Abstract

**Figure:**
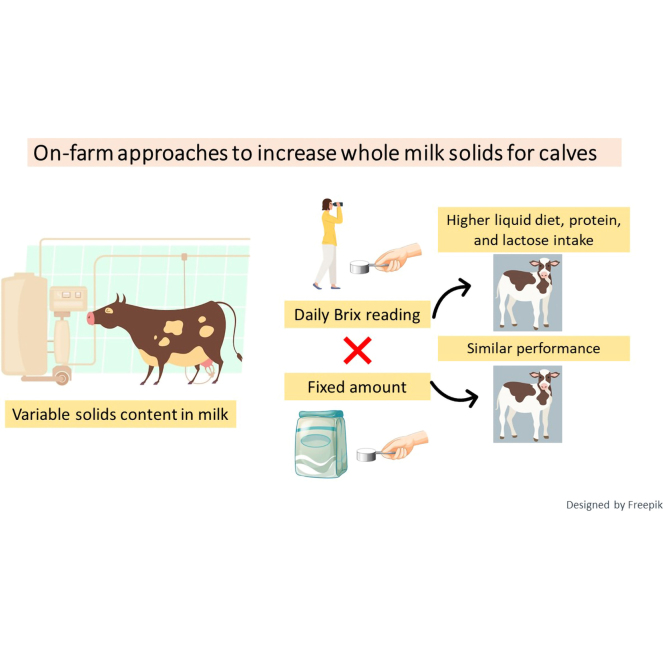
**Summary:** This study evaluated 2 methods for adjusting whole milk solids to 15% for feeding Holstein calves: a fixed-dose approach and daily adjustment using a Brix refractometer. Thirty male calves were assigned to one of the 2 methods and monitored for 56 days. Daily Brix adjustment improved liquid diet, protein, and lactose intake, and both methods resulted in similar calf performance and health. Both strategies were effective and practical for managing milk solids on dairy farms; however, using a fixed dose of a corrector or milk replacer simplifies the feeding routine. (Images from Freepik; macrovector and brgfx.)

The nutritional quality of the liquid diet, including its variations in fat, protein, and lactose content, should be carefully considered, as milk composition is influenced by multiple factors. These variations can affect TS content and, consequently, calf performance. Therefore, increasing or adjusting the TS content of the liquid diet to enhance its nutritional quality and improve nutrient intake—without altering the volume fed—may lead to more consistent and improved performance in dairy calves (Azevedo et al., 2023; Virgínio Júnior et al., 2024). Correcting and increasing the metabolizable energy and protein content of the liquid diet helps reduce variations and enhance nutrient availability, thereby promoting nutritional consistency. This, in turn, contributes to improved weight gain and overall animal health (Floren et al., 2016; Azevedo et al., 2023).

Daily monitoring of variations in the solids content of the liquid diet fed to dairy calves is a highly recommended on-farm practice, which can be performed using a Brix refractometer (Moore et al., 2009). However, conducting daily Brix readings adds an additional step to the preweaning routine, and farm staff may be reluctant to perform frequent measurements. As an alternative to daily adjustments, adding a fixed amount of solids based on the average solids content of the farm's milk may simplify calf management for farm personnel.

This study aimed to evaluate the effects of increasing the TS content of whole milk to 15% using a commercial solids corrector, either by adding a fixed amount—calculated based on the farm's mean milk solids content—or by making daily adjustments according to Brix refractometer readings, on the performance, metabolism, and health of dairy calves.

All animal procedures were performed according to the relevant guidelines and regulations, and approved according to the ethics of the Institutional Animal Care and Use Committee (protocol no. 5576050221).

The study was conducted from December 2020 to February 2021 at the Experimental Calf facilities of the Animal Science Department at Luiz de Queiroz College of Agriculture, University of São Paulo, Piracicaba, SP, Brazil.

Thirty male Holstein dairy calves from a commercial farm located approximately 50 km away were used in this study. Immediately after birth, the calves were separated from their dams, weighed, and fed with 2 doses of colostrum replacer (SSCL, Alta Genetics, Brazil; 470 g, containing 100 g of IgG/dose) within the first 2 h of life. The calves remained on the farm for 2 to 4 d, during which they were fed 4 L/d of whole milk, before being transported to the calf-rearing facility. Passive transfer was assessed by collecting a blood sample via jugular venipuncture, and total serum protein (**TSP**) was measured using a digital handheld refractometer (ITREF 200, Instrutemp, São Paulo, SP, Brazil) between 2 and 4 d of age when calves arrived at the Experimental Calf Unit. The mean TSP concentration was 5.7 ± 0.11 mg/dL, indicating adequate passive transfer for calves receiving colostrum powder (Lopez et al., 2021).

At the experimental calf facility, calves were individually housed in suspended pens (113 cm × 140 cm) with sawdust bedding inside a ventilated barn until 14 d of age. From d 15 until the end of the study (56 d), they were housed in individual wood shelters (135 cm × 100 cm × 145 cm) in an open field. The pens were cleaned daily by replacing the sawdust bed, and the shelters were moved daily to prevent fecal accumulation and to maintain a clean and dry environment. To ensure access to shade, the shelters were positioned in an east-west lateral orientation.

Calves were blocked based on birth weight and date of birth and were randomly assigned to treatments, with 2 calves per block: (1) fixed: whole milk with adjusted solids content using a fixed dose of 25 g/L (n = 15), or (2) Brix: whole milk with adjusted solids content using the daily Brix refractometer reading (n = 15). Whole milk was adjusted for 15% (150 g/L) of TS using a commercial solids corrector (Nurture Corrector, Cargill, Minneapolis, MN), which contained 245 g/kg CP, 48 g/kg ether extract (**EE**), 90 g/kg of minerals, and 3 g/kg of ADF.

Whole milk was obtained daily from the university's dairy farm and divided into 2 portions according to the volume required for each meal in the 2 treatments. Brix values were measured and recorded at each feeding. For the fixed treatment, 25 g/L of the corrector was added to achieve 15% solids, considering the average milk composition of 12.5% TS. For the Brix treatment, 10 g/L of the corrector was added for each 1% increase in Brix needed to reach 13%, corresponding to 15% TS (Moore et al., 2009). To ensure proper dilution, the corrector was gradually added to whole milk heated to 40°C and manually mixed for at least 3 min until a homogeneous solution was obtained. The liquid diet was fed to calves at 38°C across all treatments. Farm personnel were aware of the treatment groups due to the identification numbers on the feeding buckets.

Calves were fed 6 L/d of liquid diet, divided into 2 meals (0700 and 1700 h) using open buckets, and the liquid diet intake was monitored daily. All calves had ad libitum access to water and a commercial pellet calf starter (22.6% CP, 3.5% EE, 4.5% ash, 31.4% NDF, 20.9% ADF, 38.0% NFC, TDN 77.0%; Nutrimax, Salto de Pirapora, SP, Brazil). The calf starter was provided each morning, and orts were weighed using a digital scale (model 9094, Toledo do Brasil Indústria de Balanças Ltda, São Bernardo do Campo, Brazil) to calculate the daily intake. The study concluded at 56 d of age, corresponded to the preweaning period.

Liquid diet samples from each treatment were collected weekly and sent for nutrient analysis at Clínica do Leite (Piracicaba, Brazil). Additionally, SCC and total bacterial count (Table 1) were analyzed in whole milk (**WM**) before the addition of the solids corrector. Total dry matter intake (**TDMI**) was calculated daily based on the DM values of the liquid diet and starter feed.

**Table 1:** Chemical composition, SCC, and total bacteria count of the experimental liquid diets (mean ± SE)

Composition	Whole milk^1^	Brix^2^	Fixed^3^
TS, %	12.29 ± 0.36	14.83 ± 0.46	14.38 ± 0.44
Protein, %	3.38 ± 0.05	3.97 ± 0.04	3.86 ± 0.04
Fat, %	3.52 ± 0.33	3.63 ± 0.45	3.65 ± 0.39
Lactose, %	4.34 ± 0.05	6.12 ± 0.09	5.77 ± 0.09
SCC, × 10^3^/mL	424.8 ± 104.3	—	—

1 Whole milk, no adjustments.

2 Brix: whole milk with adjusted solids content (15%) using the daily Brix refractometer reading.

3 Fixed: whole milk with adjusted solids content (15%) using a fixed dose of 25 g/L.

All calves were weighed using a mechanical scale (ISO-300, Coimma Ltda., Dracena, SP, Brazil), and their body measurements (heart girth, hip width, and withers height) were taken weekly before the morning milk feed. Hip width was measured between the 2 widest points of the pelvis, and withers height was measured as the vertical distance from the ground and the highest point of the withers region. Both measurements were taken using a ruler with a centimeter scale (Carci, São Paulo, SP, Brazil). Heart girth was measured at the chest line, around the widest part of the thoracic cavity, using a tape measure with a centimeter scale (Bovitec, São Paulo, SP, Brazil). Average daily gain and feed efficiency (**FE**; kg of BW gain per kg of TDMI) were calculated for the preweaning period (1–56 d).

The diarrhea occurrence was monitored daily through visual observation of the calves' fecal consistency, as assessed using the following scoring system: (0) normal, (1) semi-formed or pasty, (2) loose, and (3) watery feces, as described by McGuirk (2008). Calves were considered to have diarrhea if the fecal score was ≥2 for 2 consecutive days and received homemade oral rehydration solution (5 g of NaCl, 25 g of dextrose, and 10 g of bicarbonate for each liter) administered 4 h after the morning feeding.

All occurrences of diarrhea, fever (rectal temperature ≥39.4°C), apathy, and other events were recorded, and treatments were performed as indicated by the veterinarian.

The blood samples were collected weekly, always 2 h after the morning feeding, via jugular venipuncture using 3 evacuated tubes (Vacuette of Brazil, Campinas, SP, Brazil) containing (1) sodium fluoride as an antiglicolytic and potassium EDTA as an anticoagulant to obtain plasma; (2) potassium EDTA, to evaluate the hematocrit; and (3) a clot activator, to obtain serum. The samples were centrifuged (Universal 320R; Hettich, Tuttlinger, Germany) at 2,000 × *g* for 25 min at 4°C. Plasma and serum were stored in a freezer (−10°C) for further analysis. The blood parameters were determined using an Automatic System for Biochemistry, model SBA, 200 (CELM, Barueri, SP, Brazil) using specific commercial kits for glucose (Ref.: 85), TSP (Ref.: 99), albumin (Ref.: 19), and creatinine (Ref.: 35) from LABTEST Diagnóstica S.A. Seventeen (Lagoa Santa, MG, Brazil). The BHB (RANBUT, Ref.: RB1007) was evaluated starting at wk 4, using a kit from RANDOX Laboratories–Life Sciences Ltd. (Crumlin, UK). The hematocrit values were determined in a capillary and centrifuged for 15 min at 12,000 × *g* at 4°C using a micro-hematocrit centrifuge (SPIN 1000, Microspin, São Paulo, Brazil). After centrifugation, the values of the hematocrit readings were expressed in percentage (%).

The sample size was calculated by the POWER procedure of SAS (version 9.0, SAS Institute Inc., Cary, NC) based on identifying a difference of 40 g/d in ADG between the 2 groups, considering a standard deviation of 20 g, a confidence level of 95%, and power of 95%. Data were screened for normality before analysis using the PROC UNIVARIATE (version 9.4, SAS Institute Inc., Cary, NC). The performance and blood parameters were analyzed as repeated measures over time using the MIXED procedure of the SAS statistical package (version 9.4, SAS Institute Inc., Cary, NC), according to the model:

Y_ijk_ = μ + T_i_ + B_j_ + W_k_ + TW_ik_ + E_ijk_,

where Y_ijk_ was the response variable, μ was the overall mean, T_i_ was the treatment effect (different protocols), B_j_ was the random block effect, W_k_ was the time effect (weeks of life), TW_ik_ was the effect of interaction between treatment and time, and E_ijk_ was the residual effect.

The covariance matrices were tested and defined according to the lowest value obtained for Akaike's information criterion corrected. The model included treatment effects, week (age of calves), and the interaction between treatment and week as fixed effects. The block effect was included in the model as a random effect. The subject of the repeated measures was the animal undergoing treatment. The method used least squares (LSMEANS) for means comparison. For all analyses, significance was declared at *P* ≤ 0.05 and a trend when 0.05 < *P* < 0.10.

No mortality occurred, and all calves were included in the analysis. Increasing TS content in WM using the Brix refractometer improved liquid diet intake (*P* < 0.01), as well as CP (*P* < 0.01) and lactose intake (*P* < 0.01; Table 2). Liquid diet fat intake was not affected by the adjustment method, nor was the starter feed intake and TDMI. All intake variables increased with age (*P* < 0.01; Table 2). Body weight at birth and weaning did not differ between treatments (Table 2). Similarly, ADG and FE also did not differ between treatments but increased with age (*P* < 0.01; Table 2). We observed a trend toward a treatment and age interaction effect (*P* < 0.10; Table 2), but no significant differences between treatments at any given time point. Body measurements were not affected by treatments or the interaction effect (Table 2).

**Table 2:** Intake and performance of dairy calves fed whole milk corrected for 15% TS using 2 approaches during the preweaning period (56 d)

Item	Treatment^1^		SEM	*P*-value^2^		
	Brix	Fixed		T	A	T × A
Liquid diet intake, g of DM/d	883.4	851.4	3.53	<0.01	0.003	0. 926
CP	236.7	228.7	1.13	<0.001	0.009	0.749
Fat	216.2	216.1	1.33	0.922	0.002	0.919
Lactose	364.6	341.6	1.43	<0.001	0.003	0.930
Starter feed intake, g of DM/d	251.9	305.3	47.53	0.426	<0.001	0.712
TDMI^3^	1,135.1	1,156.8	48.04	0.746	<0.001	0.625
Birth weight, kg	43.9	43.7	1.58	0.880	—	—
Weaning weight, kg	79.1	80.8	1.79	0.400	—	—
ADG, g/d	636.9	664.4	36.93	0.460	<0.001	0.098
Feed efficiency	0.55	0.56	0.023	0.80	<0.01	0.07
Withers height, cm	85.9	86.6	0.62	0.317	<0.001	0.826
Hip width, cm	24.1	24.0	0.31	0.817	<0.001	0.495
Heart girth, cm	88.8	89.2	1.02	0.640	<0.001	0.136

1 Brix: whole milk with adjusted solids content (15%) using the daily Brix refractometer reading (n = 15). Fixed: whole milk with adjusted solids content (15%) using a fixed dose of 25 g/L (n = 15).

2 T = treatment effect; A = age effect; T × A = treatment × age interaction effect.

3 TDMI = total dry matter intake.

The methods used to increase the TS in milk did not affect the health indicators (Table 3). The fecal score decreased as calves aged (*P* < 0.01; Table 3). Total serum protein concentration was greater for calves fed according to the fixed approach (*P* = 0.04; Table 3). Other blood parameters were not affected by the evaluated treatments. The treatment and age interaction influenced hematocrit value and creatinine concentration (*P* = 0.021 and *P* = 0.011, respectively; Table 3). Hematocrit values tended to be greater in calves from the fixed group at wk 1, whereas creatinine concentrations tended to be lower for calves from the same group at wk 2 of age. The blood parameters were also affected by the calves' age, with increasing concentrations for albumin, TSP, glucose, and BHB (*P* < 0.01; Table 3), and decreasing creatinine concentrations as calves aged (*P* < 0.01; Table 3).

**Table 3:** Health and blood parameters of dairy calves fed whole milk or whole milk corrected for 15% TS using 2 approaches during the preweaning period (56 d)

Item	Treatment^1^		SEM	*P*-value^2^		
	Brix	Fixed		T	A	T × A
Days with fever	5.9	4.8	1.30	0.429	—	—
Days with diarrhea	11.8	11.6	1.94	0.901	—	—
First diarrhea	5.3	7.1	1.34	0.200	—	—
Fecal score	0.9	0.9	0.07	0.764	<0.001	0.768
Hematocrit	25.8	26.4	0.5	0.373	0.176	0.021
Albumin, g/dL	3.1	3.1	0.03	0.716	<0.001	0.815
Total protein, g/dL	5.4	5.7	0.11	0.037	0.071	0.983
Creatinine, mg/dL	1.3	1.2	0.04	0.160	<0.001	0.011
Glucose, mg/dL	129.3	129.7	3.0	0.918	0.004	0.494
BHB, mmol/L	0.10	0.10	0.01	0.899	<0.001	0.838

1 Brix: whole milk with adjusted solids content (15%) using the daily Brix refractometer reading (n = 15). Fixed: whole milk with adjusted solids content (15%) using a fixed dose of 25 g/L (n = 15).

2 T = treatment effect; A = age effect; T × A = treatment × age interaction effect.

More intensive nutritional plans for dairy calves can enhance their growth and health during the preweaning phase (Azevedo et al., 2023). Adding commercial products, such as balancers or milk replacers, to WM to increase the TS in the liquid diet without increasing the volume offered can lead to a greater supply of nutrients, thereby improving calves' health and performance (Azevedo et al., 2016). In the present study, the addition of a solids corrector to WM resulted in a greater intake of nutrients, specifically CP and lactose.

During the preweaning period, Azevedo et al. (2016) observed that increasing TS by adding milk replacer in the WM from 12.5% to 15.0%, 17.5%, and 20.0% did not result in negative effects or differences between treatments on starter feed intake. Gelsinger et al. (2016) found a negative relationship between DMI via the liquid diet and the DMI of starter feed, which may be attributed to the limited starter feed intake capacity of young calves associated with the satiety with greater liquid diet intake and, consequently, metabolizable energy. Indeed, according to these authors, this correlation is more important when the DMI of the liquid diet is greater than 800 g/d, as observed in the present study. The difference in liquid diet DMI was 32 g/d between the Brix and fixed treatments, yet starter feed intake did not differ between these 2 groups. Azevedo et al. (2023) reported similar findings, noting that the negative effect of greater liquid diet TS on starter feed intake during the preweaning period rapidly disappears after weaning. This effect does not compromise performance, as calves on a restricted feeding exhibit greater starter feed intake without any impact on ADG (de Paula et al., 2017; Leal et al., 2021).

Using a fixed dose of the corrector to increase the TS to 15% resulted in lower daily DM liquid diet, CP, and lactose intake with no other significant differences compared with daily Brix readings. Although protein intake was greater in the Brix group, no significant improvement in structural growth was observed. Despite the greater intake of liquid diet DM, CP, and lactose associated with the Brix reading method, the differences were not substantial enough to improve performance. Furthermore, no differences in TDMI intake were observed. To our knowledge, no other study has directly compared methods to increase or correct solid content in WM to simplify feeding management. Although they are accurate, daily reading and adjustment present a challenge for commercial dairy farms adopting WM with greater solid content. Our data show that an effective alternative is to evaluate the average WM composition in the dairy farm and calculate a fixed amount of a corrector or a milk replacer to consistently increase solids in the liquid diet. The results of the present study indicate that a fixed dose approach could simplify the daily feeding routine without compromising calf performance.

Increased fluidity of the feces was observed between the second and third week of life; however, the mean fecal score did not exceed the threshold for diarrhea (≥2), indicating that the increase in TS up to 15% in liquid diet did not affect fecal consistency as a result of diarrhea. This finding is consistent with previous studies where calves were fed milk replacer (**MR**) corrected to 16% TS with 53% lactose (Virgínio Júnior et al., 2024) or MR at levels up to 20% TS in the liquid diet (Azevedo et al., 2016). The increase in the fecal score when a balancer was used in pasteurized WM can be attributed to the greater osmolality of the liquid diet, which can affect water absorption in the intestine (Glosson et al., 2015) or lead to digestive disorders (Azevedo et al., 2023). In the present study, 15% TS was adopted to provide a safe liquid diet from an osmolality standpoint, to minimize the risk of digestive disorders. Unfortunately, the osmolality of the liquid diet was not measured.

Although there was a treatment by age interaction for hematocrit, the values recorded in this study are within the normal range for this phase, with values from 20.0% to 38.0% according to Peiró et al. (2010) or 24.3% to 36.6% according to Lee et al. (2020). Most of the evaluated blood metabolites were unaffected by the different methods used to increase TS in the liquid diet. Glucose concentrations were slightly above the normal range (80 to 120 mg/dL) for calves before weaning (Reece et al., 2015). Although the overall mean difference in lactose intake was 23 g DM/d greater in the Brix group, no change was found in glucose concentration measured 2 h after ingestion of the liquid diet.

The different diets also did not affect the albumin concentrations; however, the fixed approach increased TSP, despite a decrease in the liquid diet protein intake. The concentrations of these metabolites were greater than those observed by Virgínio Júnior et al. (2024) and similar to those observed by Cezar et al. (2022). Virgínio Júnior et al. (2024) suggested that the lower albumin concentration for calves observed in their study may be attributed to a greater dilution effect in the plasma, as albumin contributes up to 75% of blood plasma osmolality. The values observed in their study were lower than the normal range of 2.75 g/dL for albumin suggested by Klinkon and Ježek (2012). Based on this information and the data in Virgínio Júnior et al. (2024), the calves in our study had a greater intake of nutrients (protein and fat).

The BHB concentrations did not differ between the liquid diets offered. The BHB concentration is a good indirect indicator of ruminal development (Cezar et al., 2022), which is associated with concentrate intake and the resulting production of short-chain fatty acids, primarily butyric and propionic (Baldwin et al., 2004). In the present study, BHB levels increased with age, the values remained below the normal range recommended by Quigley et al. (1991). However, we did not evaluate calves after 56 d.

Using a fixed dose based on average milk composition to increase solids content in WM resulted in only minor changes in nutrient intake, with no significant effect on performance, health, or the concentration of selected metabolites compared with correction based on daily Brix readings. The use of a fixed dose of a corrector or balancer to increase solids content of milk could facilitate management on dairy farms and promote the adoption of greater TS liquid diets for dairy calves.
